# The Optimization and Evaluation of Flibanserin Fast-Dissolving Oral Films

**DOI:** 10.3390/polym14204298

**Published:** 2022-10-13

**Authors:** Adel F. Alghaith, Gamal M. Mahrous, Gamal A. Shazly, Diaa Eldin Z. Zidan, Abdullah S. Alhamed, Mohammed Alqinyah, Mohammed M. Almutairi, Saeed A. Syed

**Affiliations:** 1 Department of Pharmaceutics, College of Pharmacy, King Saud University, P.O. Box 2457, Riyadh 11451, Saudi Arabia; 2 Department of Pharmacology & Toxicology, College of Pharmacy, King Saud University, P.O. Box 2457, Riyadh 11451, Saudi Arabia; 3 Department of Pharmaceutical Chemistry, College of Pharmacy, King Saud University, P.O. Box 2457, Riyadh 11451, Saudi Arabia

**Keywords:** flibanserin, hydroxypropyl cellulose, 2-hydroxy-beta-cyclodextrin, fast-dissolving oral film

## Abstract

Flibanserin (FLB) is a drug used for female hypotensive sexual desire disorder approved by the FDA in August 2015. FLB exhibits extensive hepatic first-pass metabolism and low aqueous solubility, hence poor oral bioavailability. In this study, beta hydroxypropyl cyclodextrin-FLB inclusion complexes were incorporated into orally fast dissolving films. This dosage form was expected to improve FLB aqueous solubility, which would give fast onset of action and decrease presystemic metabolism, hence improving oral bioavailability. The inclusion complex at a ratio of 1:1 was prepared by the kneading method. Differential scanning calorimetry (DSC), Fourier transform infrared spectroscopy (FTIR), and powder X-ray diffractometry (XRD) were used to confirm complex formation. The Box–Behnken design (15 different formulae of FLB fast-dissolving oral films (FLBFDOFs) were utilized for the optimization of the prepared films. The Expert Design 11 program was utilized to examine the effects of three selected factors, polymer concentration (X_1_), plasticizer concentration (X_2_), and disintegrant concentration (X_3_) on four responses: disintegration time (DT), initial dissolution rate (IDR), dissolution efficiency (*DE*), and film quality (QF). Numerical optimization was performed by minimizing disintegration time (Y1), while maximizing the initial drug dissolution rate (Y_2_), dissolution efficiency (Y_3_), and the quality factor (Y_4_). The statistical analysis showed that X_1_ has a significant positive effect on the disintegration time and a significant negative effect on IDR. While X_2_ and X_3_ produced a nonsignificant negative effect on IDR. Dissolution efficiency was maximized at the middle concentration of both X_2_ and X_3_. The best film quality was observed at the middle concentration of both X_1_ and X_2_. In addition, increasing X_3_ leads to an improvement in film quality. The optimized film cast from an aqueous solution contains hydroxypropyl cellulose (2%) as a hydrophilic film-forming agent and propylene glycol (0.8%) as a plasticizer and cross povidone (0.2%) as a disintegrant. The prepared film released 98% of FLB after 10 min and showed good physical and mechanical properties. The optimized formula showed a disintegration time of 30 s, IDR of 16.6% per minute, DE_15_ of 77.7%, and QF of 90%. This dosage form is expected to partially avoid the pre-systemic metabolism with a fast onset of action, hence improving its bioavailability that favors an advantage over conventional dosage forms.

## 1. Introduction

Female hypoactive sexual desire disorder is a premenopausal condition characterized by a decrease in sexual interest and arousal, resulting in remarkable sexual and emotional distress. FLB is considered to be the first FDA-approved drug to improve libido in premenopausal women. FLB is a centrally acting multifunctional serotonin agonist and antagonist at postsynaptic 5-hydroxytryptamine 1A (5-HT1A) and 5-HT2A receptors, respectively, which results in a net elevation in the concentration of dopamine and norepinephrine and a reduction in the concentration of serotonin [1,2]. Both dopamine and norepinephrine are important for female sexuality: their concentrations are positively correlated with improvements in sexual interest and arousal and the reduction of sexual distress [1,2].

The absolute bioavailability of FLB following oral administration is around 33% due to high hepatic first-pass metabolism and its low water solubility [3,4]. FLB has a high protein-binding affinity in serum (98%), primarily for albumin. FLB is extensively metabolized by cytochrome P450 3A4 (CYP3A4) and to a lesser extent by CYP2C19 to pharmacologically inactive metabolites, which are primarily excreted in urine and feces. Therefore, improving the solubility and developing and using an alternative route of administration are clinically warranted to improve the bioavailability of FLB.

The inclusion complex is a commonly used method of improving the water solubility of poorly soluble compounds by using cyclodextrins (CDs) [5]. CDs are cyclic oligosaccharides composed of α-(1→4)-linked α-d-glucopyranose. The central core of cyclic oligosaccharides is hydrophobic in nature, while its outer shell is hydrophilic [6]. Different drugs form complexes with CDs via the incorporation of hydrophobic drugs into the hydrophobic central cavity of the CDs. The main advantage of the inclusion complexes is the improvement of drug solubility, stability, and bioavailability. Furthermore, there are additional multiple applications for inclusion complexes including masking the unfavorable taste or smell of certain drug formulations [7]. Based on glucose molecule numbers, CDs are classified into three types: six-glucopyranose units forming α-CD, seven glucopyranose units forming Β-CD, and eight glucopyranose units forming γ-CD [8]. These types of CDs vary in their physical properties as exemplified by the low solubility of Β-CD s compared to other types. The safety profile and water solubility of Β-CD s can be improved by conjugating several hydroxyl groups of a Β-CD with propylene oxides, forming modified hydroxypropyl-beta-cyclodextrin (HP-Β-CD) [9].

Orally fast-dissolving film is a novel dosage form that is intended to quickly disintegrate and absorb upon contacting the highly vascular and permeable oral mucosal tissues [10]. This dosage form is generally composed of plasticizers, sweeteners, salivary stimulants, colorants, surfactants, and hydrophilic polymers [10]. The active pharmaceutical ingredient is incorporated into the matrix of hydrophilic polymers to enhance drug dissolution and release from the film upon contacting saliva. This drug delivery system provides a promising route of administration for drugs with suboptimal oral absorption and bioavailability induced by a high hepatic first-pass effect [11]. Orally dissolving films increase the efficacy of the active pharmaceutical ingredient by dissolving in the oral cavity in a short duration upon contacting less amount of saliva as compared to dissolving tablets [12]. It was reported that the in vitro disintegration time of oral polymeric films, determined by the cell method, correlated with the in vivo disintegration time [13].

Orally fast-dissolving films have been widely used to deliver many drugs, which allow for gastrointestinal tract absorption after swallowing saliva containing the dissolved drug or pregastric absorption in the oral mucosa to bypass the digestive system and hepatic first-pass metabolism. Multiple studies have reported that oral mucosal (buccal and sublingual) absorption of fast-dissolving films enhances drug bioavailability via bypassing the first-pass metabolism and avoiding pre-systemic clearance [14,15,16,17,18,19].

These dosage forms have superiority over traditional tablets and capsules, as they have enormously improved patient acceptance and compliance, especially in pediatric, geriatric, dysphagic, and psychiatric patients [10]. These dosage forms are more convenient for patients as they do not require chewing or water for administration. Previously published data showed that FLB was formulated as nasal gel [4] and buccal tablets [20]. In a previous study, the complexation of FLB with HP-β-CD improved its dissolution and permeation [20]. However, to the best of our knowledge, no data were published on FLB oral film. The aim of this study was to prepare fast-dissolving oral films containing FLB complexed with HP-β-CD. FLBFOD film is expected to give fast onset of action and partially avoid the first pass metabolism, hence improving its bioavailability that favors an advantage over conventional dosage forms.

## 2. Materials

FLB was purchased from Qingdao Sigma Chemical Co., Ltd. (Qingdao, China). Hydroxyl propyl cellulose (HPC), low viscosity grade, and propylene glycol (PG) were purchased from BDH Co. (Poole, UK). Tartaric acid was obtained from Serva GmbH & Co. (Heidelberg, Germany). Beta hydroxypropyl cyclodextrin (HP-β-CD) was purchased from Signet Chemical Corporation Pvt. Ltd. (Mumbai, India). The other chemicals used were of analytical grade.

## 3. Methods

### 3.1. Spectrophotometric Scanning of FLB

To determine the wavelength of maximum absorption (λmax), a specified concentration of FLB was scanned spectrophotometrically at 200–400 nm using a UV mini 1240 spectrophotometer (Shimadzu, Duisburg, Germany). UV spectrophotometric scanning of FLB solutions in presence of polymer solutions in phosphate buffer pH 6.8 was also investigated to detect any interference.

### 3.2. FLB Calibration Curve Construction

FLB was measured at λ 244 nm and a calibration curve was obtained by accurately weighing 10 mgs of FLB and dissolving it in 100 mL of dilute hydrochloric acid. Dilutions were made in phosphate buffer (pH 6.8) to obtain different concentrations corresponding to 25, 50, 75, 100, 150, 200, and 400 ug/mL of the drug.

### 3.3. Preparation of Solid Inclusion Complexes

Inclusion complexes were prepared in a molar ratio of 1:1 for FLB and HP-β-CD

#### 3.3.1. Physical Mixture

FLB and HP-β-CD physical mixture (PM) in a 1:1 molar ratio of FLB and HP-β-CD was prepared by simple trituration in porcelain mortar for five minutes.

#### 3.3.2. Kneading Method

Complex in a 1:1 molar ratio of FLB and HP-β-CD was prepared as previously reported [14]. In brief, FLB and HP-β-CD mixture in a molar ratio of 1:1 was triturated in a porcelain mortar, then wetted with drops of acetone/water mixture and kneaded for additional 30 min. The obtained mass was then dried at 40 °C for 48 h.

### 3.4. Characterization of the Inclusion Complex

#### 3.4.1. Differential Scanning Calorimetry (DSC)

To investigate any possible interactions between FLB and HP-β-CD, DSC thermogram were obtained for FLB, HP β-CD, their physical mixtures, as well as the complex of FLB (1:1) with HP β-CD using PerkinElmer DSC8000 (PerkinElmer Inc., Waltham, MA, USA). A sample of 5 to 7 mg was weighed into aluminum pans and tightly closed. DSC was run at a heating rate of 10 °C/min over a temperature range of 25–300 °C. Endothermic peak temperature and heat of fusion were determined using the software.

#### 3.4.2. Fourier Transforms Infrared Spectroscopy (FTIR)

Potassium bromide disks were prepared using 5 mg of FLB, HP-β-CD; their physical mixture, or kneaded complex. Spectra were recorded in scanning range 400–4000 cm^−1^, on FT/IR-4100 type A (Bruker Co., Billerica, MA, USA).

#### 3.4.3. Powder X-ray Diffractometry (XRD)

Samples of FLB and its complex were performed using a RIGAKU diffractometer (RIGAKU, Tokyo, Japan). The XRD patterns were obtained by scanning from 4° to 100° with 2° variation.

### 3.5. Preparation of FLB Fast-Dissolving Oral Films (FLBFDOFs)

**Figure 1:**
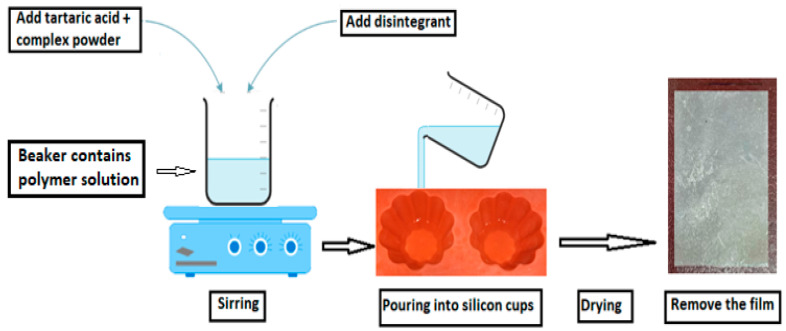
Schematic diagram for FLBFDOFs preparation.

FLBFDOFs composed of film-forming polymer (HPC) in addition to a plasticizer (PG) were prepared by the solvent casting method [21]. The specified amount of polymer was dispersed over the required amount of distilled water in a beaker and allowed to swell. Then, the polymer solution was stirred using a magnetic stirrer until dissolved completely g. Thereafter, the complex, tartaric acid, and propylene glycol were weighed and added followed by stirring until complete dissolution. Finally, Crospovidone was added and mixed thoroughly. The polymeric solution was poured into clean and dry circular silicon cups of 2 cm^2^ area, and each film contains 10 mg of the FLB. The films were dried in an oven (Mermmert U—Schwabach, Germany) at 40.0 °C for 24 h. Thereafter, the films were carefully removed and stored in a desiccator at room temperature after wrapping them with aluminum foil. A schematic diagram for FLBOFDs preparation is shown in Figure 1. A list of formulations and their components is shown in Table 1.

### 3.6. Evaluation of the Prepared FLB Films

#### 3.6.1. Film Thickness

The thickness was measured at five different locations of the whole intact film using a micrometer (Moore and Wright, Sheffield, UK). The test was performed in triplicate, and the average thickness ± SD was recorded [22].

#### 3.6.2. Film Quality

The quality of the produced films was assessed based on the following criteria: (1) flexibility (endurance > 30 times), (2) spreadability during pouring onto the casting cups, (3) stickiness, (4) easy to peel off casting cups, and (5) appearance. Each criterion was given 20% of the total value [12].

#### 3.6.3. In Vitro Disintegration

A USP disintegrator (PTZ-S Pharma Test Apparatebau AG, Hainburg, Germany) was used to calculate FLBFDOF disintegration time. For each formula, 3 films were tested using phosphate buffer pH 6.8 maintained at 37 °C ± 2 °C as a disintegration medium and the time for complete disintegration was recorded [22].

#### 3.6.4. FLBFDOF Content

In a 50 mL volumetric flask, FLBFDOF was dissolved using ethanol, then sonicated for 10 min to ensure the complete dissolving of the FLB. Then, 1 mL was diluted with 10 mL of ethanol, and the sample was filtered through a 0.45 μm filter. Finally, FLB content was determined using HPLC assay.

#### 3.6.5. HPLC assay Conditions

Chromatography was performed on a qualified Waters UPLC Acquity system, having a binary pump, vacuum degasser, a sample manager equipped with a robotic auto sampler system, and a photo diode array detector. Empower software 2 was used for data acquisition and processing. The used column was Waters BEH C18 UPLC column (1.7 µm, 2.1 × 100 mm). The system was from Waters (Milford, MA, USA).

Instrumental Conditions: The used wavelength was 221 nm, which keeps a resolution of 1.2 nm. The flow rate was 0.2 mL/min, and mobile phases A and B were run at a ratio of 50% each, the column temperature and auto sampler were set at ambient temperature, and the retention time of the FLB peak was observed at ~1.665 min.

#### 3.6.6. In Vitro Release Studies

For dissolution experiments, USP apparatus 2 (Caleva Ltd., Model 85T, Sturminster Newton, UK) was used. In each flask, 300 mL of phosphate buffer pH 6.8 was equilibrated to 37 ± 0.5 °C. An accurately weighed film of each formula was added to each flask and operated at 75 rpm. Samples were withdrawn at 5, 10, 15, and 30 min time intervals. For each formula, 3 films were tested, and the absorbance was measured at 244 nm. The cumulative percent released of FLB was determined and plotted as a function of time.

### 3.7. Response Surface Methodology for Optimization of FLBFDOFs

The Expert design 11 program was utilized to determine the effect of the selected factors on various responses. Table 2 shows the experimental design with different levels of independent factors. The 15 runs of the experiment were evaluated for the disintegration time (Y_1_), initial dissolution rate (Y_2_), dissolution efficiency (Y_3_), and quality factor (Y_4_).

## 4. Results and Discussion

### 4.1. FLB Spectrophotometric Assay

**Figure 2:**
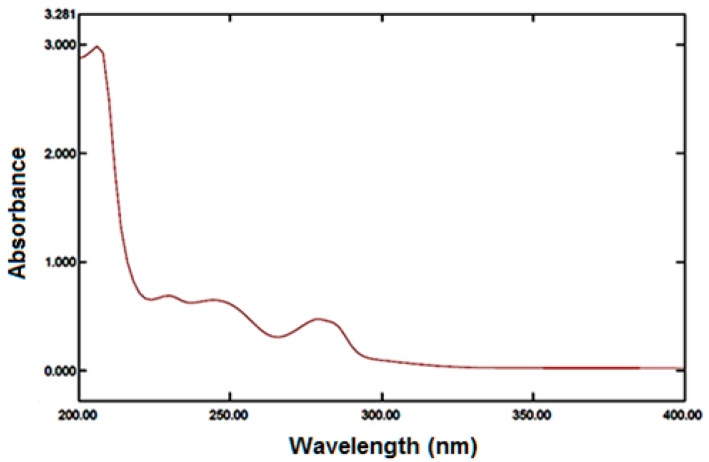
UV Spectrophotometric scans for FLB solution.

**Figure 3:**
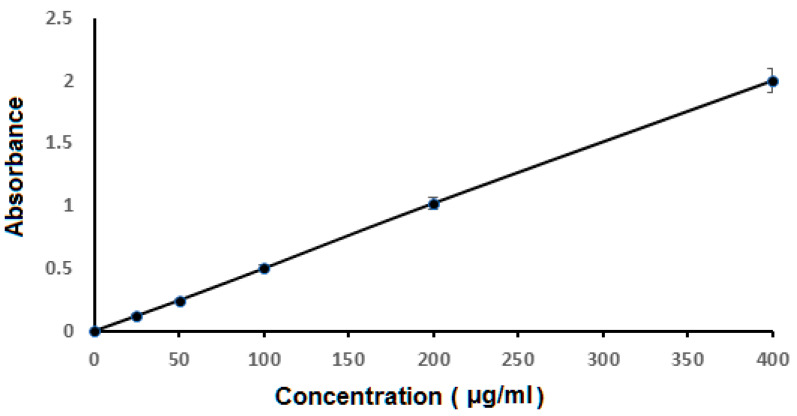
Calibration curve for FLB at 244 nm.

FLB spectrophotometric scanning showed maximum absorbance at 244 nm (Figure 2). However, there was no interference at 224 nm when other excipients were applied. A calibration curve was plotted that demonstrated a linear relationship between absorbance and FLB concentration in the range of 25–400 μg /mL in phosphate buffer of pH 6.8, as shown in Figure 3. The regression equation is y = 0:005 x, and the regression coefficient was 0. 9999.

### 4.2. DSC Studies

**Figure 4:**
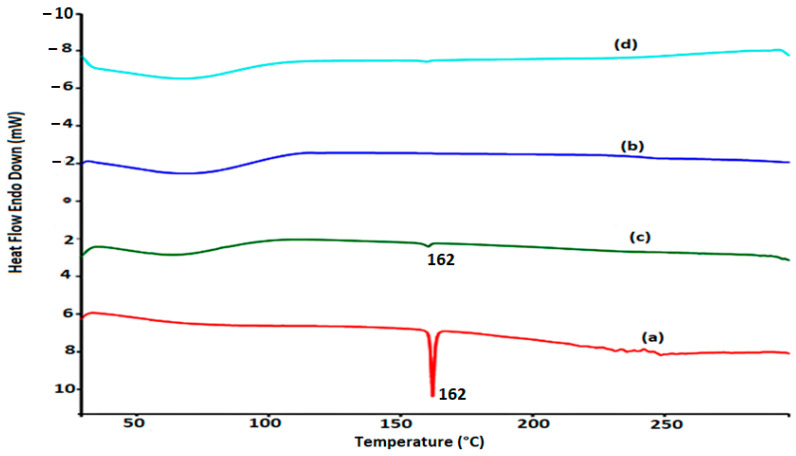
DSC thermograms of FLB (a), HP-BCD (b), physical mixture (c), complex (d).

The DSC thermogram of pure FLB (Figure 4) displayed a melting endotherm at 162.0 °C. However, the thermogram of pure HP-Β-CD (Figure 4) exhibited a broad endotherm ranging from about 70 °C to 110 °C, as a result of the presence of water [23]. The 1:1 physical mixture of FLB and HP-Β-CD (Figure 4) showed the same melting peak of FLB without a sharp peak. This phenomenon occurs as a consequence of the decrease in FLB and the presence of the HP-Β-CD carrier (dilution effect). The results obtained with the 1:1 HP-Β-CD-FLB inclusion complex (Figure 4) revealed a significant decrease in the intensity of the FLB melting peak, thus could indicate that the inclusion complex was formed properly. These results confirm our previously reported data [20].

### 4.3. FTIR Studies

**Figure 5:**
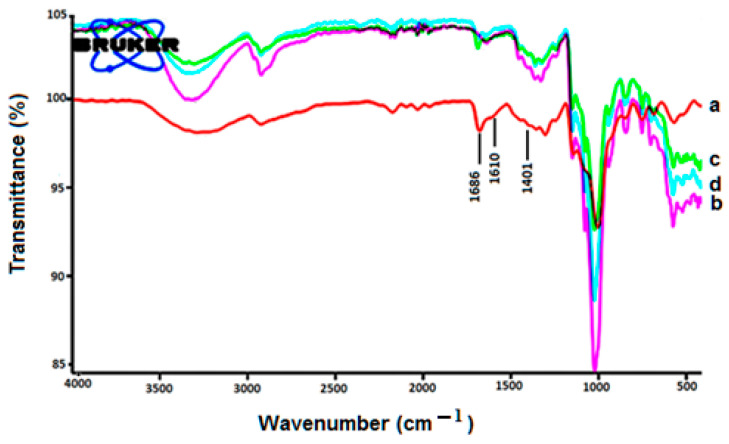
FTIR of FLB (a), HP-β-CD (b), physical mixture (c), complex (d).

Figure 5 shows the IR spectrum of FLB. Assigned peaks detected at 1686 and 1610 cm^−1^ and in the region of 1401–1448 cm^−1^, reflecting, respectively, -C=O stretching, N-H bending, and aromatic C=C. Figure 5 demonstrates the FTIR spectrum of HP-Β-CD in which O-H stretching vibrations provide a broad absorption band at 3423 cm^−1^. Interestingly, intense peaks were observed in the C-H bond at 2931 cm^−1^ assigned to its stretching vibrations. However, bands between the ranges of 1384 and 1460 cm^−1^ were assigned to the bending vibrations of CH2 and CH3. Furthermore, our study also showed that physical mixture IR spectra displayed differential variations marked by decreased peak intensities compared with the IR spectra obtained from pure FLB and HP-Β-CD (Figure 5). The bands’ intensities in the range of 1681–1306 cm^−1^, assigned to the stretching vibrations of C=O and the bending from the NAH aromatic ring, were markedly altered by the formation of the complex with HP-Β-CD (Figure 5). Furthermore, our study showed not only quantitative but also qualitative differences in the IR spectra of pure FLB, HP-Β-CD, and the physical mixture. These differences between the IR spectra of pure compounds and the binary systems, particularly the inclusion compound, suggest that there are interactions between the two compounds in the binary systems, which strongly support the findings that were previously reported [20,24].

### 4.4. X-ray Diffractometry

**Figure 6:**
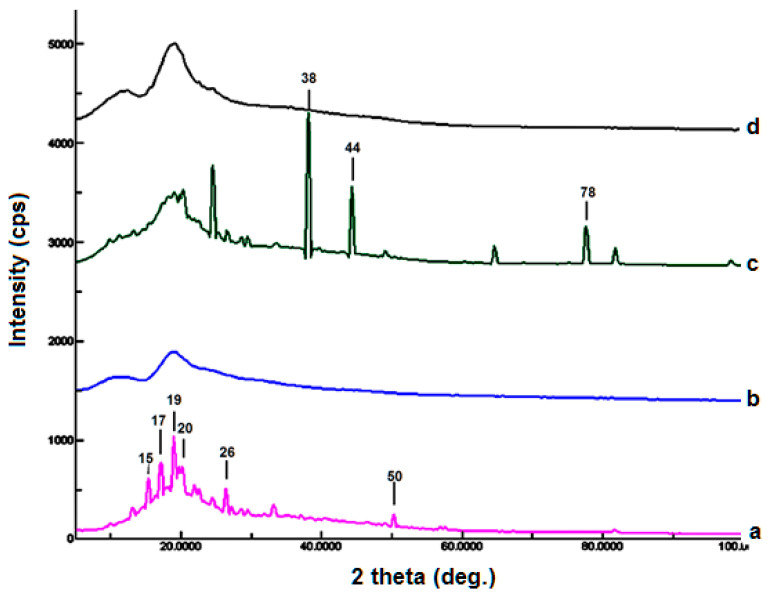
Powder X-ray of FLB (a), HP-BCD (b), physical mixture (c), complex (d).

Data shows the XRD patterns of FLB and inclusion complexes prepared using the kneading method (Figure 6). FLB displayed a crystalline pattern with characteristic peaks at 15.3°, 17.1°, 19°, 20.2°, 26.4°, and 50.2°. On the other hand, HP-Β-CD showed an amorphous pattern. In addition, XRD demonstrated crystalline peaks of FLB, which indicates that the physical trituration technique is not effectively useful in the preparation of inclusion complexes with FLB. Interestingly, some new diffraction peaks were observed in the physical mixture at 38°, 44°, and 78°. Subsequently, this could be attributed to the interaction between uncomplexed drug and HP-β-CD resulting in a new crystalline form [24]. However, the 1:1 HP-β-CD-FLB inclusion complex obtained using the kneading approach clearly demonstrated an amorphous appearance, which indicates that the HP-β-CD-FLB inclusion complex was successfully, formed using the kneading approach. These results support our previously reported findings [20,25]. Taken together, the results of DSC, FTIR, and XRD confirm the complex formation, which are consistent with our previously reported data [20].

### 4.5. FLB Film Formation

The main scope of this study was to optimize formulation parameters to develop FLBFDOFs. There are numerous characteristics of a successful film that should be achieved. These characteristics include excellent content uniformity, adequate mechanical strength, and integrity, as well as fast disintegration upon hydration. In addition, many factors control suitable mechanical strength and film integrity, including optimizing the polymer concentration, the plasticizer concentration, and optimizing the disintegrant concentration [12,13]. In our study, the effects of three independent factors (X_1_: HPC polymer (%), X_2_: plasticizer concentration (PG) (%), and X_3_: disintegrant concentration (%) on the critical dependent variables (responses) such as disintegration time, initial dissolution rate, dissolution efficiency, and film quality were studied to optimize FLB film formulation.

### 4.6. FLB Orally Fast-Dissolving Film (FLBFDOF) Evaluation

Since the use of thin-film oral dosage forms relies on their disintegration in the saliva of the oral cavity, the final film must necessarily be water-soluble. To prepare a thin film formulation that is water-soluble, the polymer must be water-soluble with low molecular weight and excellent film-forming capacity [26]. The polymer used in this study was HPC, in addition to tartaric acid as a saliva stimulating agent, PG as a plasticizer, and CPV as a super disintegrant.

The physical inspection of the prepared films showed successfully obtained films with good quality. The average weights of films ranged from 0.13 to 0.282 g depending on the composition of each formula (Table 1). Increasing polymer concentration from 1 to 3% showed an increase in the weight and consequently in film thickness, which ranged from 0.14 to 0.35 mm (Table 1). In addition, all formulations showed fast disintegration time, which ranged from 30 to 50 s (Table 3). The mean time dissolution from all formulations was fast, and the initial dissolution rate calculated from the first 10 min ranged from 10 to 18%/min (Table 3). The dissolution efficiency at 15 min was calculated using Equation (1) and the results exhibited a range of 51.4 to 85% (Table 3):

(1) $$DE\;=\;\frac{{ʃ}_{t_{2}}^{t_{1}}y\cdot dt}{y100\times \left(t_{2}-t_{2}\right)}\times 100$$

where *y* is the percentage of the dissolved product. *DE* is the area under the dissolution curve between time points *t*_1_ and *t*_2_ expressed as a percentage of the curve at maximum dissolution, *y*_100_, over the same time period.

The effects of the dependent factors: polymer concentration, plasticizer concentration, and disintegrant concentration on the selected responses: disintegration time, initial dissolution rate, dissolution efficiency, and film quality are discussed in detail below under Statistical Analysis and Summary of Fit.

### 4.7. Determination of FLB Content in the Prepared Films

**Figure 7:**
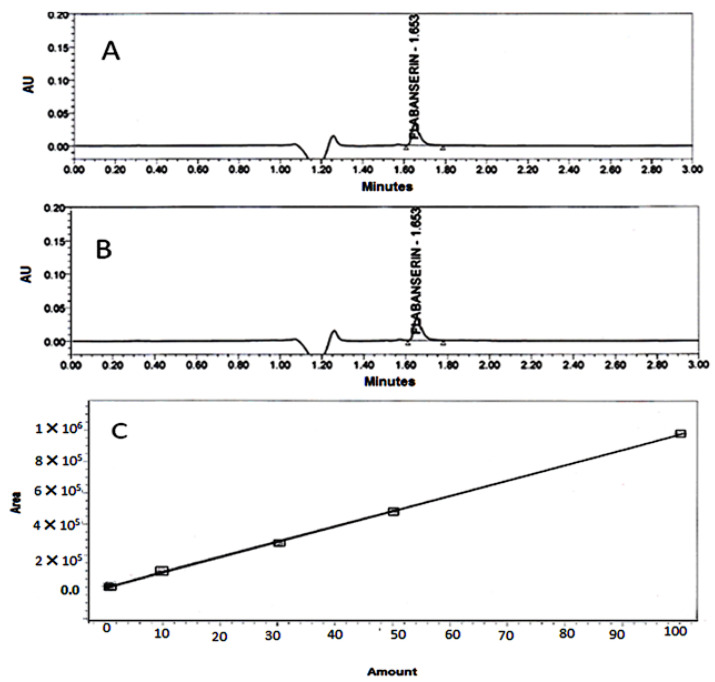
FLP HPLC chromatograms and calibration curve, (**A**): pure FLB; (**B**): FLB film, (**C**): FLB calibration curve.

FLB content in the prepared films was determined using both UV spectrophotometric and UPLC methods. It was found that there was a correlation between the UV spectrophotometric and UPLC assays as shown in Figure 3 and Figure 7. Therefore, UV spectrophotometric assay was used in the drug release studies. HPLC assay was used to determine the content and to check the stability of FLB during film preparation. It was found that the FLB content ranged from 95 to 105% of the theoretical content in all film formulations which is complying with the pharmacopoeial guidelines USP30-NF2 [27]. In addition, the single peak obtained (Figure 7) indicates the stability of the drug with no degradation during film preparation [28].

### 4.8. Response Surface Methodology for Optimization of FLBFDOFs

The Expert design11 program was utilized to determine the effect of the selected factors on various responses (as shown in Table 2). Box–Behnken design was performed for the optimization of FLBFDOFs with minimum disintegration time, maximum initial dissolution rate, maximum dissolution efficiency, and the highest quality. The design (Box–Behnken) of experiments with observed and predicted results is presented in Table 3.

### 4.9. Statistical Analysis and Summary of Fit

To investigate the effects of control factors on the responses, different statistical approaches, including linear, quadratic, interactive, and polynomial terms, were performed. The coefficients of model terms and associated *p*-values for Y_1_–Y_4_ are summarized in Table 4. Significant responses of factors were considered if *p*-value was less than 0.1 (*p* < 0.1). To simplify the regression model, the non-significant terms (*p* > 0.1) were not considered.

A positive or negative coefficient indicated an increase or decrease in the corresponding response, respectively, to the increase or decrease in the level of the factor or factors involved in that term. For experimental design validity, analysis of variance (ANOVA), R^2^, and adjusted R^2^ were used (summarized in Table 4). High R^2^ and adjusted R^2^ indicated good data fit for the investigated responses. In addition, the lack of fit *p*-values for Y_1_–Y_4_ were 0.36, 0.582, 3.8, and 0.51, respectively, which were greater than 0.1 for all responses, suggesting that model errors for the selected models were not significant [29].

### 4.10. Effect of Independent Variables on the In Vitro Disintegration Time (Y_1_)

**Figure 8:**
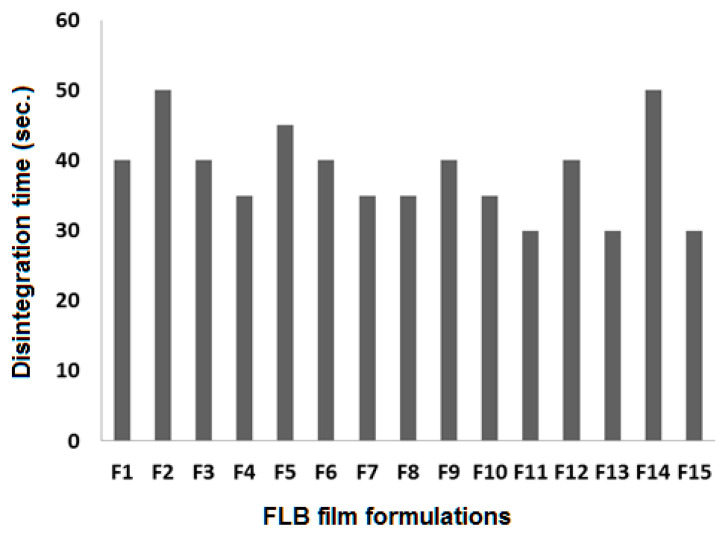
Disintegration times for FLBFDOF formulation.

The disintegration time is a critical parameter that plays an important role in the pattern of release and consequent absorption of the drug through the biological membranes. The fast disintegration of oral-dissolving films is important to ensure rapid breakdown into smaller fragments to yield the largest possible surface area that is applicable for dissolution media [30]. FLBFDOFs formulations showed variations in disintegration times ranging from 30 s (F13&F15) to 50 s (F2&F14). The results are presented as a histogram in Figure 8.

To investigate the effects of control factors on the disintegration time, results were subjected to statistical analysis. According to the highest correlation coefficient (R^2^), the data shown in Table 4 demonstrated that disintegration values were fitted to the quadratic model. Moreover, there was an acceptable agreement between the predicted and adjusted R^2^, indicating that the model is valid. In addition, adequate precision for the response was greater than 3, demonstrating an adequate signal-to-noise ratio, which indicates the selected model’s suitability to explore the design space.

Analysis of variance (ANOVA) for the disintegration time confirmed the significance of the quadratic model as depicted by its F-value of 16.73. The lack of fit *p*-value of 0.36 showed a non-significant lack of fit compared to pure error, ensuring data fitting to the proposed model. The equation representing the quadratic model was generated in terms of coded factors as follows:

Y_1_ = 30 + 3.75 X_1_ − 3.13 X_2_ − 1.88 X_3_ − 2.5 X_1_X_2_ − 2.3 X_1_X_3_ + 1.5 X_2_X_3_ + 6.88 X_1_^2^ + 3.13 X_2_^2^+ 5.63 X_3_^2^ (2)

**Figure 9:**
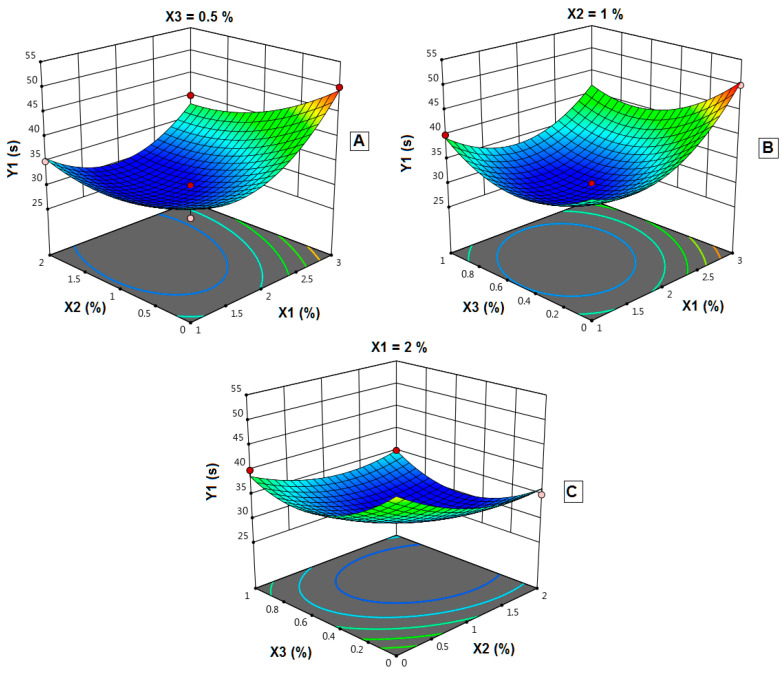
Effect of independent variables on the in-vitro disintegration time (Y_1_). (**A**): Effect of polymer concentration (X_1_) and plasticizer concentration (X_2_) on Y_1_ at 0.5% X_3_. (**B**): Effect of polymer concentration (X_1_) and disintegrant concentration (X_3_) on Y_1_ at 1% X_2_. (**C**): Effect of plasticizer concentration (X_2_) and disintegrant concentration (X_3_) on Y_1_ at 2% X_1_.

The statistical analysis showed that the polymer concentration (X_1_) has a major effect on the disintegration time. Increasing X_1_ leads to a significant increase in the disintegration time (*p*-value 0.0028). Plasticizer concentration (X_2_) and disintegrant concentration(X_3_) have a negative impact on disintegration time. In which the increase in both X_2_ and X_3_ lead to a decrease in disintegration time. Figure 9A–C showed the response surface plots for the studied variables’ effects on disintegration time (Y_1_). Interestingly, the disintegration time significantly increases with increasing X_1_ (Figure 9A). Increasing X_3_ from 0 to 1% showed a maximum decrease in disintegration time at 0.4–0.6% while keeping X_2_ at 1% (Figure 9B). At X_1_ of 2%, X_2_ and X_3_ exhibited a non-significant effect on disintegration time (Figure 9C).

### 4.11. Influence of Independent Variables on the In-Vitro Initial Dissolution Rate (Y_2_)

**Figure 10:**
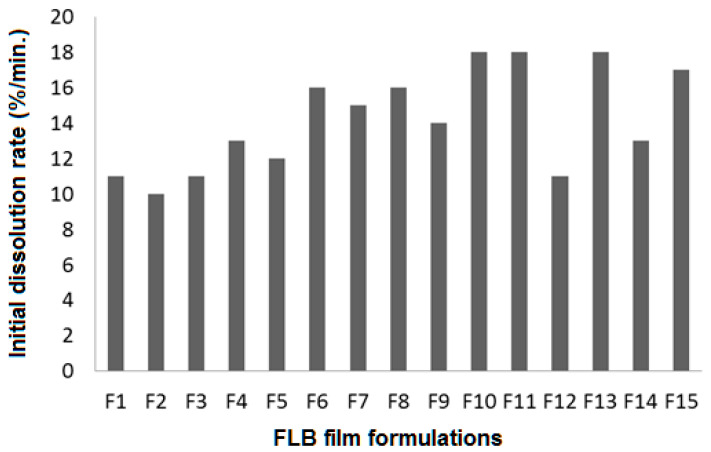
IDR (%) for FLBFDOF formulations.

The initial dissolution rate is a fundamental parameter that demonstrated a significant influence on the release pattern and consequently absorption of the drug through the biological membranes [31]. FLBFDOF formulations showed variations in initial dissolution rate ranging from 10–18 s (Figure 10). It was observed that the higher polymer concentration (3%) showed the lowest IDR. Based on the highest correlation coefficient (R^2^), the data of IRD fitted to the quadratic model, as shown in Table 4. In addition, there was a reasonable agreement between R^2^ and adjusted R^2^ indicating the validity of the model. Furthermore, adequate precision for the response was greater than 3. Taken together, this indicates an adequate signal-to-noise ratio, which suggests the suitability of the selected model to explore the design space.

Analysis of variance (ANOVA) for the initial dissolution rate confirmed the significance of the quadratic model as depicted by its F-value of 4.37. The lack of fit *p*-value of 0.04 implied a significant lack of fit relative to the pure error.

The equation representing the quadratic model was generated in terms of coded factors as follows:

Y_2_ = 17.67–1.75 X_1_ + 0.375 X_2_ + 0.375 X_3_ − 0.25 X1X_2_ − 1.25 X_1_X_3_ − 1.5 X_2_X_3_ − 2.83 X_1_^2^ − 1.08 X_2_^2^ − 2.58 X_3_^2^ (3)

The statistical analysis revealed that X_1_ has a significant negative effect on IDR, while X_2_ and X_3_ showed a non-significant positive effect at a polymer concentration of 2%.

**Figure 11:**
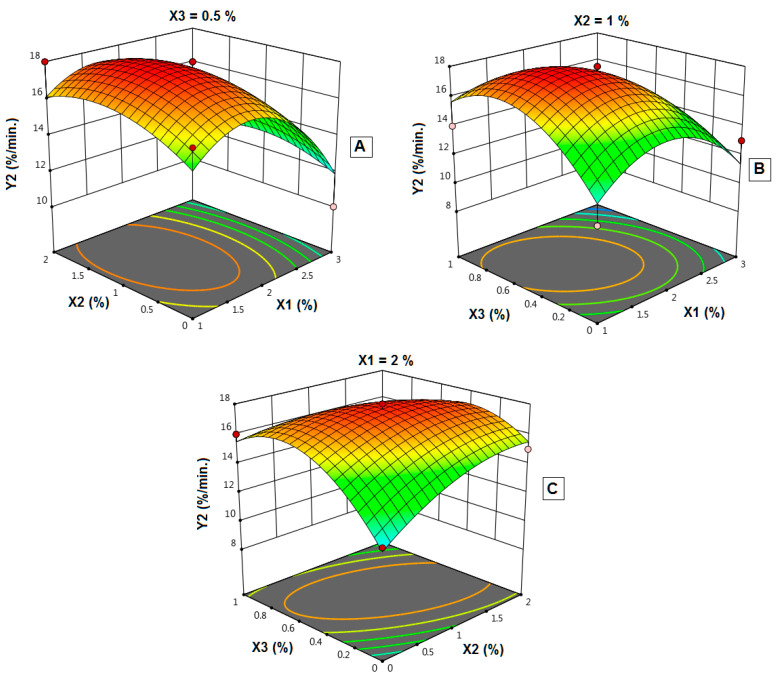
Influence of independent variables on the in-vitro initial dissolution rate (Y_2_). (**A**) Effect of polymer concentration (X_1_) and plasticizer concentration (X_2_) on Y_2_ at 0.5% X_3_. (**B**) Effect of polymer concentration (X_1_) and disintegrant concentration (X_3_) on Y_2_ at 1% X_2_. (**C**) Effect of plasticizer concentration (X_2_) and disintegrant concentration (X_3_) on Y_2_ at 2% X_1_.

Figure 11 illustrates the impacts of the independent variables’ interaction on the IDR (Y_2_). It shows the response surface plots for the effects of the studied variables on IDR. Interestingly, the IDR significantly decreased with increasing polymer concentration from 2% to 3% (Figure 11A). At 1% plasticizer concentration along with, increasing both polymer concentration from 1% to 2% and disintegrant concentration to 0.5%. There was an increase in IDR (Figure 11B). In addition, at polymer concentration of 2%, X_2_ and X_3_ showed the highest IDR at the middle concentration for both (Figure 11C).

### 4.12. Influence of Independent Variables on the Dissolution Efficiency (Y_3_)

Dissolution is the process by which a substance forms a solution. Several properties, including drug dissolution rate, determine the oral bioavailability of a drug. In terms of drug absorption from the gastrointestinal tract, the rate-limiting step in this process includes the release and dissociation of the drug from the dosage form. Pharmaceutical compounds with an aqueous solubility of less than 100 μg/mL often have a limited dissociation-absorption pattern [32]. FLB is a class II biopharmaceutical classification system (BCS) and its complexation with HP-B-CD resulted in an increase in its aqueous solubility [20].

**Figure 12:**
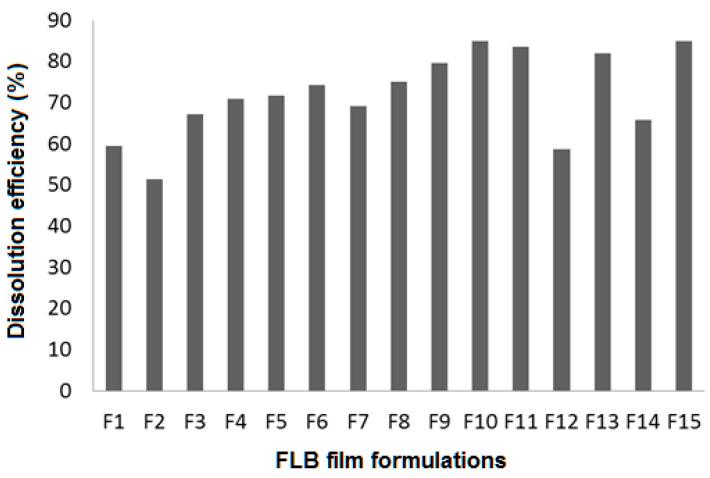
*DE* (%) for FLBFDOFs formulations.

The in vitro dissolution profiles of FLB from the prepared FLBFDOFs in the dissolution medium pH 6.8 were performed. To calculate dissolution efficiency after 15 min (DE_15_%), Equation (1) was used. The DE_15_% of FLBFDOF formulations ranged from 51.4 to 85% (Figure 12). F15 showed the highest *DE*, and F2 showed the lowest. To explore the effect of the three dependent factors on *DE*, statistical analysis was applied.

Based on the highest correlation coefficient (R^2^), the data of dissolution efficiency (Y_3_) fitted to the quadratic model, as shown in Table 4. In addition, there was a reasonable agreement between the actual R^2^ and adjusted R^2^ indicating the validity of the model. Furthermore, adequate precision for the response was greater than 3, indicating an adequate signal-to-noise ratio, which implies the suitability of the selected model for exploring the design space.

Analysis of variance (ANOVA) for the dissolution efficiency confirmed the significance of the quadratic model as depicted by its F-value of 4.99. The lack of fit *p*-value of 0.044 implied a significant lack of fit relative to the pure error. The model proposes the following polynomial equation for dissolution efficiency

Y_3_ = 83.17 − 8.93 X_1_ + 1.42 X_2_ + 1.3 X_3_ − 0.7 X_1_X_2_ − 4.65 X_1_X_3_ − 0.2 X_2_X_3_ − 9.6 X_1_^2^ − 6.06 X_2_^2^ − 5.61 X_3_^2^ (4)

As shown in Table 4, the *p*-value of 0.0578 shows a significant effect of the corresponding factors on dissolution efficiency. In this case, X_1_ and X_1_^2^ are significant model terms, i.e., the polymer concentration has a negative effect on *DE*. Increasing polymer concentration from 2 to 3% resulted in a decrease in *DE*.

**Figure 13:**
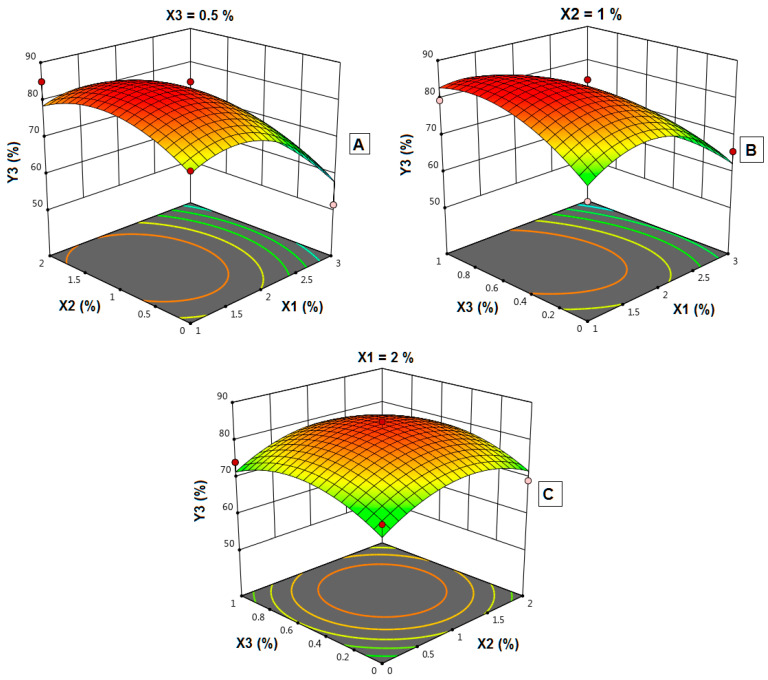
Effect of independent variables on dissolution efficiency (%) (Y_3_). (**A**): Effect of polymer concentration (X_1_) and plasticizer concentration (X_2_) on Y_3_ at 0.5% X_3_. (**B**): Effect of polymer concentration (X_1_) and disintegrant concentration (X_3_) on Y_3_ at 1% X_2_. (**C**): Effect of plasticizer concentration (X_2_) and disintegrant concentration (X_3_) on Y_3_ at 2% X_1_.

The combined effects of X_1_, X_2_, and X_3_ on dissolution efficiency (*DE*) are shown in Figure 13A–C. As X_1_ increased from 1 to 2%, *DE* increased. By further increase in X_1_ from 2 to 3%, *DE* decreased significantly (*p* = 0.008). *DE* was maximized at the middle concentration of both X_2_ and X_3_; however, this effect was not statically significant.

### 4.13. Influence of Independent Variables on the Quality Factor (Y_4_)

Theoretically, a successful film should exhibit adequate mechanical strength and integrity. Film integrity can be controlled by increasing the polymer concentration, which will increase the film thickness and optimize the plasticizer concentration, whereas increasing the disintegrant concentration could decrease the film quality [33]. The quality of the produced films was assessed based on the following criteria: flexibility, spreadability during pouring onto the casting cups, stickiness, ease of peeling off casting cups, and appearance. Each criterion was given 20% of the total value, and a total numerical value of 100% was calculated as the quality factor (Y_4_).

**Figure 14:**
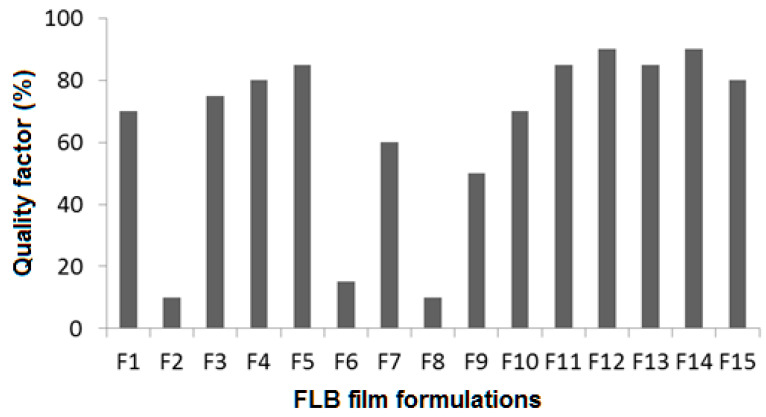
QF (%) for FLBFDOFs formulations.

Figure 14 shows the results of estimated QF for prepared FLBFDOFs. F2, F6, and F8 which contain no plasticizer showed the lowest QF values. This shows the importance of the plasticizer in the formula, however, increasing its concentration more than the optimum level could lead to a decrease in film quality (F7&F10). It was reported that plasticizer is a vital ingredient of fast dissolving films. Plasticizer helps to improve the flexibility of the strip and reduces the brittleness of the films. It significantly improves the film-forming properties by reducing the glass transition temperature of the polymer [34].

To explore the contribution and the interaction of the studded independent factors on the film quality and based on the highest correlation coefficient (R^2^), the data of quality factor (Y_4_) fitted to the quadratic model, as shown in Table 4. In addition, there was an acceptable agreement between the R^2^ and adjusted R^2^. This indicates the validity of the model. In addition, adequate precision for the response was greater than 3, indicating an adequate signal-to-noise ratio, which implies the suitability of the selected model for exploring the design space.

Analysis of variance (ANOVA) for the quality factor confirmed the significance of the quadratic model as depicted by its F-value of 3.64. The lack of fit *p*-value of 0.0192 implied a significant lack of fit relative to the pure error.

The model proposes the following polynomial equation for the quality factor:

Y_4_ = 83.33 + 6.87 X_1_ + 22.50 X_2_ − 11.88 X_3_ + 5.00 X_1_X_2_ + 1.2 X_1_X_3_ + 22.5 X_2_X_3_ − 13.54 X_1_^2^ − 24.79 X_2_^2^ + 1.46 X_3_^2^ (5)

**Figure 15:**
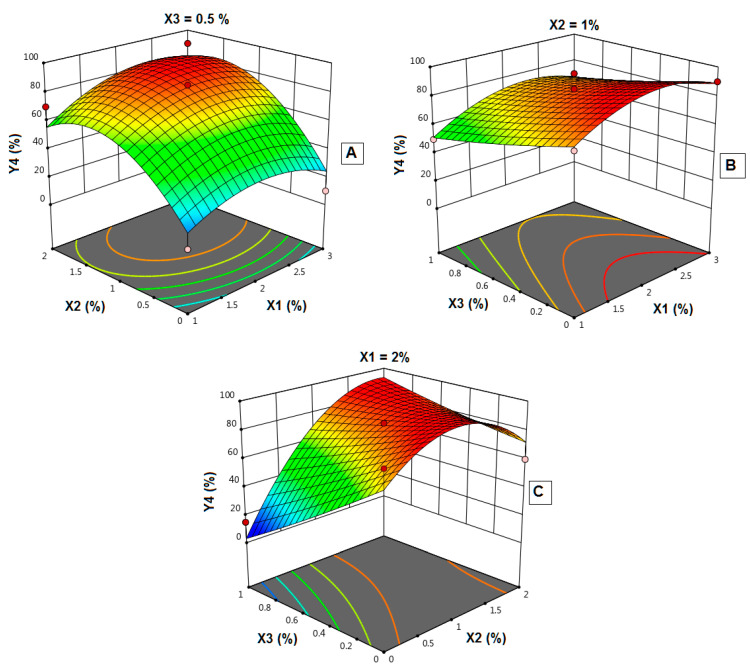
Influence of independent variables on the quality factor (Y_4_). (**A**) Effect of polymer concentration (X_1_) and plasticizer concentration (X_2_) on Y_4_ at 0.5% X_3_. (**B**) Effect of polymer concentration (X_1_) and disintegrant concentration (X_3_) on Y_4_ at 1% X_2_. (**C**) Effect of plasticizer concentration (X_2_) and disintegrant concentration (X_3_) on Y4 at 2% X_1_ (**A**–**C**).

As shown in Table 4, the *p*-value of 0.0989 represents a significant effect of the corresponding factors on the quality factor. In this case, X_2_, X_2_X_3_, X_2_^2^, and X_3_^2^ are significant model terms. This might be explained as the influence of all the factors contributing to film properties in various formulations. Effects of polymer concentration, plasticizer concentration, and disintegrant concentration on the quality factor are shown in Figure 15A–C. The best film quality was observed at the middle concentration of both X_1_ and X_2_. In addition, increasing X_3_ leads to a decrease in film quality.

#### Optimization

**Figure 16:**
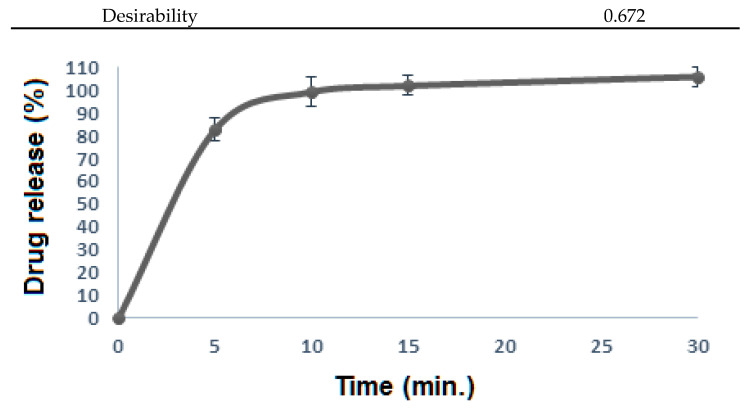
FLB release from the optimized film formula.

The use of DoE programs to optimize the different formulation parameters is well established [4,21]. To integrate the obtained results from different parameters, the Expert design 11 program was utilized. Numerical optimization was performed by minimizing the disintegration time (Y_1_) while maximizing the initial drug dissolution rate (Y_2_), dissolution efficiency, (Y_3_), and the quality factor (Y_4_). The predicted optimum formula was obtained and prepared (donated as F16 in Table 1). The predicted and observed values of the responses for the optimized FLBFDOFs formula are displayed in Table 5. Figure 16 shows the release profile of the optimized formula. The prepared FLB films showed comparable observed responses with the predicted ones regarding disintegration time, the initial drug dissolution rate, dissolution efficiency, and film quality, indicating the validity and predictability of the employed experimental design as well as the production of FLBFDOFs have optimal properties.

## 5. Conclusions

Our research study succeeded at developing and validating orally dissolving films containing 10 mg FLB. The D-optimal multifactorial design of the experiment applied in this study significantly helped to understand the effect of independent variables (polymer concentration, plasticizer concentration, and disintegrant concentration) on four responses (disintegration time, the initial drug dissolution rate, dissolution efficiency, and the film quality). The analysis of variance demonstrated that all the independent variables had an impact on all responses. The selected model revealed a high level of reliability and enabled the identification of design space that produced FDOF formulations with optimal properties. The optimized film cast from an aqueous solution contains hydroxypropyl cellulose (2%) as a hydrophilic film-forming agent and propylene glycol (0.8%) as a plasticizer and cross povidone (0.2%) as a disintegrant. Interestingly, the developed film successfully released 98% of FLB in 10 min with good physical and mechanical properties. The optimized formula showed a disintegration time of 30 s, IDR of 16.6% per minute, *DE* of 77.7%, and QF of 90%. This new FLB formulation has superiority over traditional tablets and capsules, as they have enormously improved patient acceptance and compliance, especially in geriatric, dysphagic, and psychiatric patients. In addition, this dosage form is expected to partially avoid the pre-systemic metabolism with a fast onset of action, hence improving its bioavailability that favors an advantage over conventional dosage forms.

## Figures and Tables

**Table 1:** Composition of different formulae of FLBFDOFs.

Formulation	Ingredients (% w/w in the Dispersions)					Average Film Weight (g)	Average Thickness (mm)
	HPC	Propylene Glycol	Crospovidone	Tartaric Acid	FLB Complex (Contains 10 mg FLB)		
F1	3	1	1	0.5	1	0.277 ± 0.003	0.37 ± 0.01
F2	3	0	0.5	0.5	1	0.203 ± 0.006	0.3 ± 0.01
F3	1	1	0	0.5	1	0.134 ± 0.004	0.1 ± 0.01
F4	2	2	1	0.5	1	0.234 ± 0.009	0.22 ± 0.01
F5	2	0	0	0.5	1	0.16 ± 0.001	0.13 ± 0.01
F6	2	0	1	0.5	1	0.207 ± 0.006	0.21 ± 0.01
F7	2	2	0	0.5	1	0.193 ± 0.015	0.163 ± 0.006
F8	1	0	0.5	0.5	1	0.13 ± 0.01	0.14 ± 0.01
F9	1	1	1	0.5	1	0.205 ± 0.005	0.24 ± 0.01
F10	1	2	0.5	0.5	1	0.165 ± 0.007	0.17 ± 0.01
F11	2	1	0.5	0.5	1	0.21 ± 0.006	0.303 ± 0.005
F12	3	2	0.5	0.5	1	0.282 ± 0.004	0.35 ± 0.01
F13	2	1	0.5	0.5	1	0.213 ± 0.006	0.3 ± 0.01
F14	3	1	0	0.5	1	0.257 ± 0.012	0.25 ± 0.01
F15	2	1	0.5	0.5	1	0.257 ± 0.004	0.203 ± 0.058
Optimized (F16)	2	0.8	0.2	0.5	1	0.214 ± 0.002	0.213 ± 0.006

**Table 2:** Variables in Box-Behnken design for FLBFDOFs.

**Independent Variables, Factor**	**Low (−1)**	**Middle (0)**	**High (1)**
X1: HPC Polymer (%)	1	2	3
X2: Plasticizer concentration (PG) (%)	0	1	2
X3: Disintegrant concentration (%)	0	0.5	1
**Dependent Variables, Response**			
Y1: Disintegration time (s)			
Y2: initial dissolution rate (%)			
Y3: Dissolution efficiency (%)			
Y4: quality factor (%)			

**Table 3:** Design (Box–Behnken) of experiments with results.

Runs	Independent Variable			Dependent Variables							
				Observed Value				Predicted Value			
	X_1_	X_2_	X_3_	Y_1_	Y_2_	Y_3_	Y_4_	Y_1_	Y_2_	Y_3_	Y_4_
1	3	1	1	40	11.00	59.50	70	41.88	9.63	55.68	67.50
2	3	0	0.5	50	10.00	51.40	10	49.38	11.88	57.85	24.38
3	1	1	0	40	11.00	67.10	75	38.13	12.38	70.93	77.50
4	2	2	1	35	13.00	70.90	80	35.00	13.25	74.03	93.13
5	2	0	0	45	12.00	71.70	85	45.00	11.75	68.58	71.88
6	2	0	1	40	16.00	74.20	15	38.75	15.50	71.58	13.13
7	2	2	0	35	15.00	69.20	60	36.25	15.50	71.83	71.88
8	1	0	0.5	35	16.00	75.00	10	36.88	14.88	74.30	20.63
9	1	1	1	40	14.00	79.50	50	39.38	15.63	82.83	51.25
10	1	2	0.5	35	18.00	85.00	70	35.63	16.13	78.55	55.63
11	2	1	0.5	30	18.00	82.50	85	30.00	17.67	83.17	83.33
12	3	2	0.5	40	11.00	58.60	90	38.13	12.13	59.30	79.38
13	2	1	0.5	30	18.00	82.00	85	30.00	17.67	83.17	83.33
14	3	1	0	50	13.00	65.70	90	50.63	11.37	62.38	88.75
15	2	1	0.5	30	17.00	85.00	80	30.00	17.67	83.17	83.33

X_1_ = HPC%, X2 = PG%, X_3_ = Disintegrant concentration (%), Y_1_ = Disintegration time (s), Y_2_ = IDR (%), Y_3_ = Dissolution efficiency (%), Y_4_ = QF (%).

**Table 4:** ANOVA analysis for the selected models for different responses of FLBFDOFs.

Response	Model	Sequential *p*-Value	Lack of Fit *p*-Value	R^2^	Adjusted R^2^	Adequate Precision	Significant Terms	F Value
Disintegration time (Y_1_)	Quadratic	0.0032	0.36	0.96	0.91	13.04	X_1_, X_1_^2^, X_2_^2^, X_3_^2^	16.73
Initial dissolution rate (Y_2_)	Quadratic	0.073	0.04	0.8	0.5	4.935	X_1_, X_1_^2^_,_ X_3_^2^	4.37
Dissolution efficiency (Y_3_)	Quadratic	0.0578	0.044	0.88	0.664	5.7	X_1,_ X_1_^2^	4.99
Quality factor (Y_4_)	Quadratic	0.0989	0.019	0.81	0.6926	6.8415	X_2_, X_3_, X_2,_ X_3_, X_2_^2^	3.64

**Table 5:** Results of the optimized versus the predicted formula.

	Optimized Formula (F16)	Predicted Formula
Disintegration time (s)	30	33
IDR % per minute	16.60	15.00
DE_15_ %	77.70	80.00
QF %	90.00	90.00
Desirability		0.672

## Data Availability

Data will make available on request.
